# Whole-genome sequencing of matched primary and metastatic hepatocellular carcinomas

**DOI:** 10.1186/1755-8794-7-2

**Published:** 2014-01-09

**Authors:** Limei Ouyang, Jeeyun Lee, Cheol-Keun Park, Mao Mao, Yujian Shi, Zhuolin Gong, Hancheng Zheng, Yingrui Li, Yonggang Zhao, Guangbiao Wang, Huiling Fu, Jhingook Kim, Ho Yeong Lim

**Affiliations:** 1 BGI-Shenzhen, Beishan Industrial Zone, Beishan Road, Shenzhen, Yantian 518083, People’s Republic of China; 2 Division of Hematology-Oncology, Department of Medicine, Samsung Medical Center, Sungkyunkwan University School of Medicine, 50 Irwon-dong, Gangnam-gu, Seoul 135-710, Korea; 3 Department of Pathology, Samsung Medical Center, Sungkyunkwan University School of Medicine, Seoul 135-710, Korea; 4 Oncology Research Unit, Pfizer Inc., San Diego CA 92121, USA; 5 Department of Thoracic and Cardiovascular Surgery, Samsung Medical Center, Sungkyunkwan University School of Medicine, 50 Irwon-dong, Gangnam-gu, Seoul 135-710, Korea

**Keywords:** Cancer, Hepatocellular carcinomas (HCC), Lung metastasis, Somatic, Next-generation sequencing (NGS)

## Abstract

**Background:**

To gain biological insights into lung metastases from hepatocellular carcinoma (HCC), we compared the whole-genome sequencing profiles of primary HCC and paired lung metastases.

**Methods:**

We used whole-genome sequencing at 33X-43X coverage to profile somatic mutations in primary HCC (HBV+) and metachronous lung metastases (> 2 years interval).

**Results:**

In total, 5,027-13,961 and 5,275-12,624 somatic single-nucleotide variants (SNVs) were detected in primary HCC and lung metastases, respectively. Generally, 38.88-78.49% of SNVs detected in metastases were present in primary tumors. We identified 65–221 structural variations (SVs) in primary tumors and 60–232 SVs in metastases. Comparison of these SVs shows very similar and largely overlapped mutated segments between primary and metastatic tumors. Copy number alterations between primary and metastatic pairs were also found to be closely related. Together, these preservations in genomic profiles from liver primary tumors to metachronous lung metastases indicate that the genomic features during tumorigenesis may be retained during metastasis.

**Conclusions:**

We found very similar genomic alterations between primary and metastatic tumors, with a few mutations found specifically in lung metastases, which may explain the clinical observation that both primary and metastatic tumors are usually sensitive or resistant to the same systemic treatments.

## Background

Hepatocellular carcinoma (HCC) is one of the most common cancers worldwide with an estimated 500,000-1,000,000 new cases per year [1]. It is also one of the few cancers with an increased incidence rate [2-4]. Although curative resection or liver transplant is widely adopted as treatment for HCC [5], a majority of HCC patients are not amenable to surgical resection due to the extensive intrahepatic tumor burden or limited donor resources. In nature, HCC is an invasive tumor and is metastasized hematogenously and lymphogenously to other organs, even after its local recurrence. The most common organs of distant metastases include lungs, lymph nodes, bone, and brain, in particular, with the lung metastasis occurring in 18-60% of HCC cases [6]. While extrahepatic metastases are known to occur in HCC patients with an advanced intrahepatic tumor stage [6,7], the prognosis in patients with extrahepatic metastases is extremely poor. To improve the prognosis for HCC metastases, we aim to investigate the genetic signatures of HCC through unbiased next-generation sequencing (NGS) in this whole genome-scale systematic study.

Recently, whole genome sequencing of metastatic acral melanomas and matched primary tumor and adjacent normal tissues showed high similarity of global gene copy number alterations, loss of heterozygosity and single nucleotide variation in primary and metastasis tissues [8]. Meanwhile, *de novo* nonsynonymous coding single-nucleotide variants (SNVs) were identified in metastatic lesions from acral melanomas [8]. In addition, recent studies that identified the clonal populations by sequencing the genomes of 7 pancreatic cancer metastases helped elucidate the mechanisms of distant metastases [9,10]. Herein, we sequenced the whole genomes of 4 paired samples (primary tumor, metachronous lung metastases) obtained from HCC patients. These selected patients were treatment naïve at the time of primary HCC surgery and were later diagnosed with lung metastatic nodules more than 2 years after the first surgery. We compared the genetic landscape between primary liver and metastatic lung tumor tissues and performed an in-depth investigation of the genetic alterations to uncover potential biomarkers for tumor progression and metastasis.

## Results

### Case description

Four patients who were pathologically diagnosed of HCC and lung metastases were included in this study.

#### Case #1(441)

A 50-year old man presented with a 9-cm sized mass at right hepatic lobe. The patient was a HBV carrier and his liver function was of Child-Pugh classification of A. The patient underwent right liver lobectomy when freshly frozen tissue of primary tumor and blood DNA was collected. The pathologic review of the surgical specimen revealed Edmonson grade II, tumor necrosis of 10%, microvascular tumor emboli and chronic active hepatitis with bridging fibrosis in background liver. Three years later, the patient developed a 2-cm sized single metastatic nodule in posterior basal segment of right lower lobe. The patient underwent right lower lobectomy and the pathologic diagnosis was metastatic hepatocellular carcinoma. Currently, the patient is in remission without recurrence since lung metastatectomy for more than 5 years.

#### Case #2(DD59)

A 46 year-old man presented with a 5.2 × 4.4 cm massive HCC involving S6 and S7. The patient was a hepatitis B carrier. The patient underwent right hemi-hepatectomy (tissue procurement of primary tumor). The pathologic features were as follows, Edmonson grade II, 0% necrosis, intrahepatic metastasis, segmental portal vein microinvasion and negative resection margin. Three months later, the patient developed small recurrent tumor in left lateral segment of left lobe of liver and underwent radiofrequency ablation and 6 cycles of transcatheter arterial chemoembolization (TACE) thereafter. Shortly after the 6^th^ TACE, two nodules were detected in right lung. The patient underwent video-assisted thoracoscopic surgery wedge resection of right upper apical segment, posterior segment and RLL lobectomy. The pathologic diagnosis was metastatic hepatocellular carcinoma. Fresh frozen tissues were collected during the surgery at this time. Seven months later the patient developed another 2 cm lung metastatic nodule in right middle lobe and underwent metastatectomy. The patient is currently in remission for 4 years.

#### Case #3(D473)

A 43-year old male presented with an 11-cm sized HCC-B (hepatitis B) and underwent right hepatectomy. The pathologic examination revealed Edmonson grade II HCC with 10% necrosis, no portal vein invasion with mild portoperiportal inflammation in background liver due to chronic hepatitis B. Four months following the surgical resection, a newly developed enhancing nodule in S3 of liver was detected which was subsequently treated with 5 cycles of TACE. Two years from the initial hepatic resection, a 1 cm nodule in left upper lobe was detected which was surgically removed with wedge resection. The tumor specimen was confirmed of metastatic HCC in lung and freshly frozen tissue was collected at this time. The patient underwent repeated multiple pulmonary metastatectomies for recurrent metastatases. The patient still has slowly progressive, multiple lung metastases which were refractory to sorafenib and sunitinib. The patient died of disease ten months later after failing to treatment due to disease progression.

#### Case #4(D430)

A 51-year old male had a 6.6 c m sized liver mass and was diagnosed of HCC-B. The patient underwent S6 segmentectomy and the pathologic examination revealed Edmondson grade II, 60% necrosis, no microvessel or portal vein invasion and no intrahepatic metastasis. Two years later, a 14 cm solitary lung nodule was detected in left lower lobe superior segment which was surgically resected. Thirty months later, the patient developed multiple HCC nodules and received ten 10 cycles of TACE. The patient is still undergoing periodic TACE for multiple HCCs at the time of this writing. The core information is marked with specific notes (Additional file 1: Table S1 and Additional file 2: Figure S1).

### Whole genome sequencing

**Figure 1:**
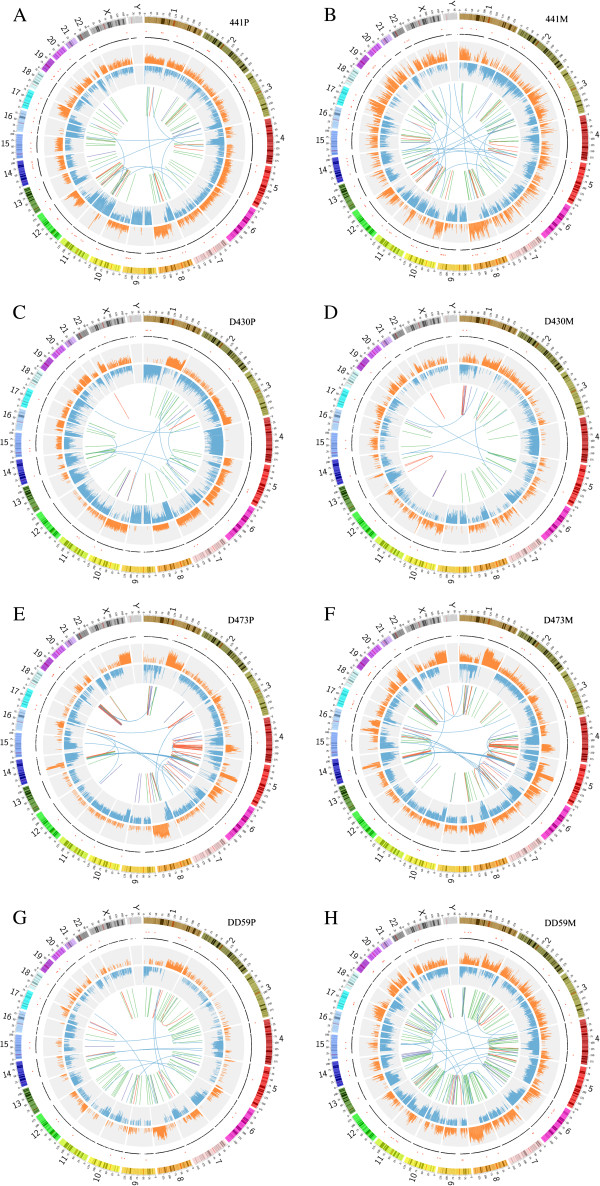
**Circos plots for the 4 primary tumors and their matched metastases.** Outer panels represent somatic SNVs with nonsynonymous and splice mutations in red, others are in black. Somatic copy number alterations are shown in inner panels (orange histogram, duplications; blue histogram, deletions). Structural variations are depicted as links in the interior of the plots. Blue link, inter-chromosomal translocations; red link, intra-chromosomal translocations; purple link, insertions; green link, deletions. Plots of **A** to **H** show the variants of primary tumor and metastasis from each of the 4 cases 441, D430, D473 and DD59 with “P” for primary and “M” for metastasis.

Using the paired-end sequencing strategy, we simultaneously generated 98.90Gb-129.65Gb sequence data from normal liver tissues, primary HCC tumors, and lung metastases of the 4 patients described above, with the corresponding haploid genome coverage of 33–43 fold at high coverage >99% (Additional file 1: Table S2A). The widespread somatic alterations were identified throughout the genome, from somatic single nucleotide variants (SNVs) and small insertions or deletions (indels) to structural variations (SVs) (Figure 1 and Additional file 1: Table S2F). We next depicted the whole-genome alterations as Circos plots, which give visual demonstrations of metastasis-specific genetic alterations (Figure 1). Subsequently, we performed an in-depth analysis of the mutations, copy number alterations, and structure variations.

### Mutation analysis

**Figure 2:**
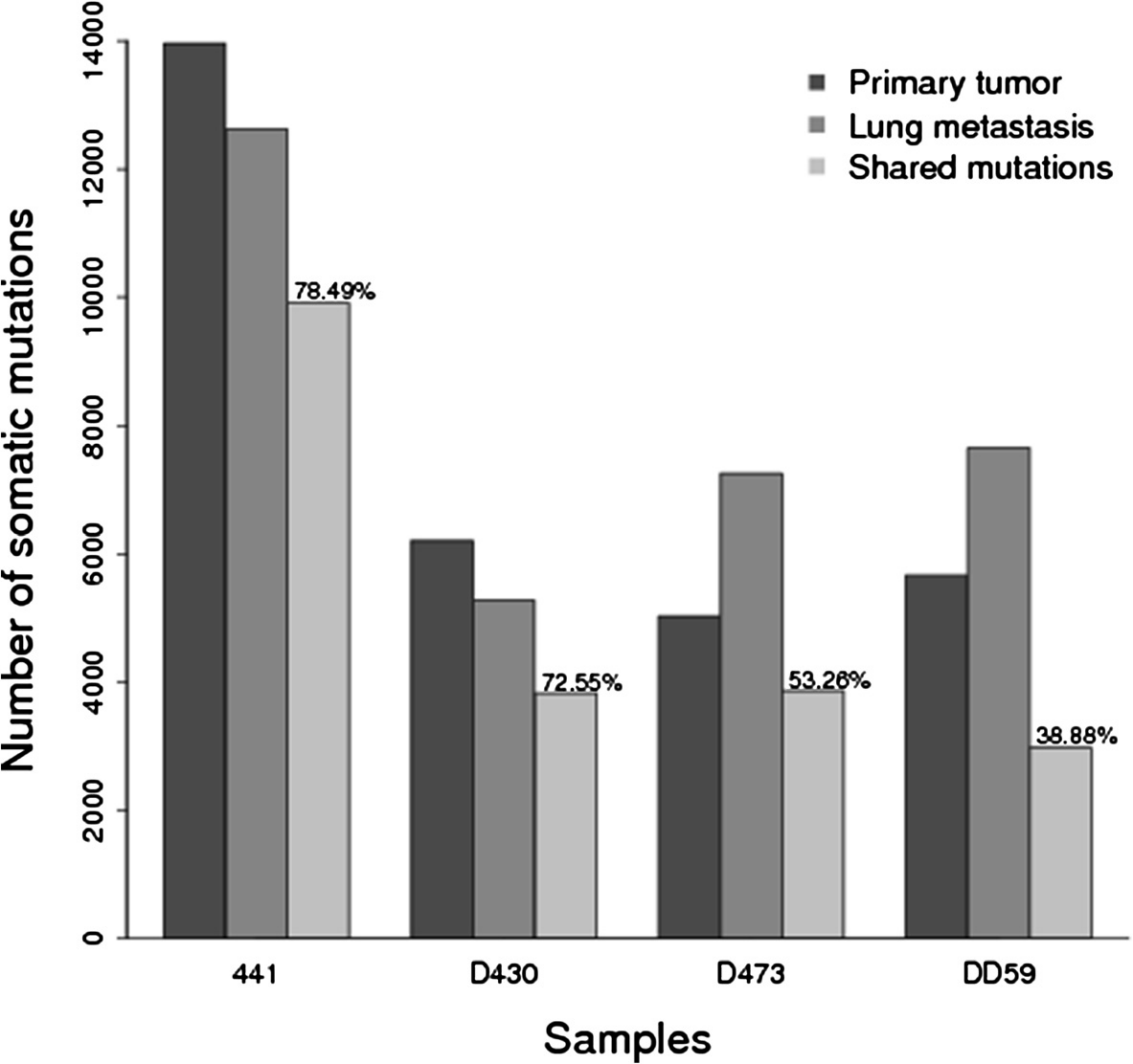
**The number of somatic mutations detected in primary tumors and metastases.** The percentage of mutations shared by metastases and primary tumors was calculated, and the mutations are summarized into 3 groups: primary tumors, metastases, and shared mutations.

Alignments to the reference genome with opened gaps were performed using BWA (http://bio-bwa.sourceforge.net/bwa.shtml), and SOAPsnv [11] was used to query SNVs from tumors against matched normal tissues. 3.40-8.63% of total SNVs were detected in dbSNP (dbSNP132). These known germline SNPs were excluded from downstream analysis. The SNVs were annotated to RefGene and summarized according to their genetic features (Table 1). In total, 5,027-13,961 somatic SNVs were detected in HCC primary tumors and 5,275-12,624 were in lung metastatic tumors. In addition, most SNVs detected in lung metastases were also present in primary tumors, ranging from 38.88% to 78.49% across 4 individuals, suggesting that the major genetic characteristics of tumors might be preserved during metastasis (Figure 2).

**Table 1:** Summary of somatic SNVs

**Categories**	**441**		**D430**		**D473**		**DD59**	
	**Primary tumor**	**Metastasis**	**Primary tumor**	**Metastasis**	**Primary tumor**	**Metastasis**	**Primary tumor**	**Metastasis**
# of somatic SNVs	13,961	12,624	6,208	5,275	5,027	7,251	5,666	7,652
Synonymous	24	26	19	13	13	11	5	13
Nonsense	4	3	1	0	3	3	0	4
Missense	74	69	23	18	22	36	33	45
Splice	5	5	0	0	0	0	1	2
UTR	84	76	43	30	37	44	44	57
Intron	4,216	3,826	1,911	1,667	1,680	2,354	1,878	2,543
Intergenic	9,554	8,619	4,211	3,547	3,272	4,803	3,705	4,988

### Mutation spectrum

**Figure 3:**
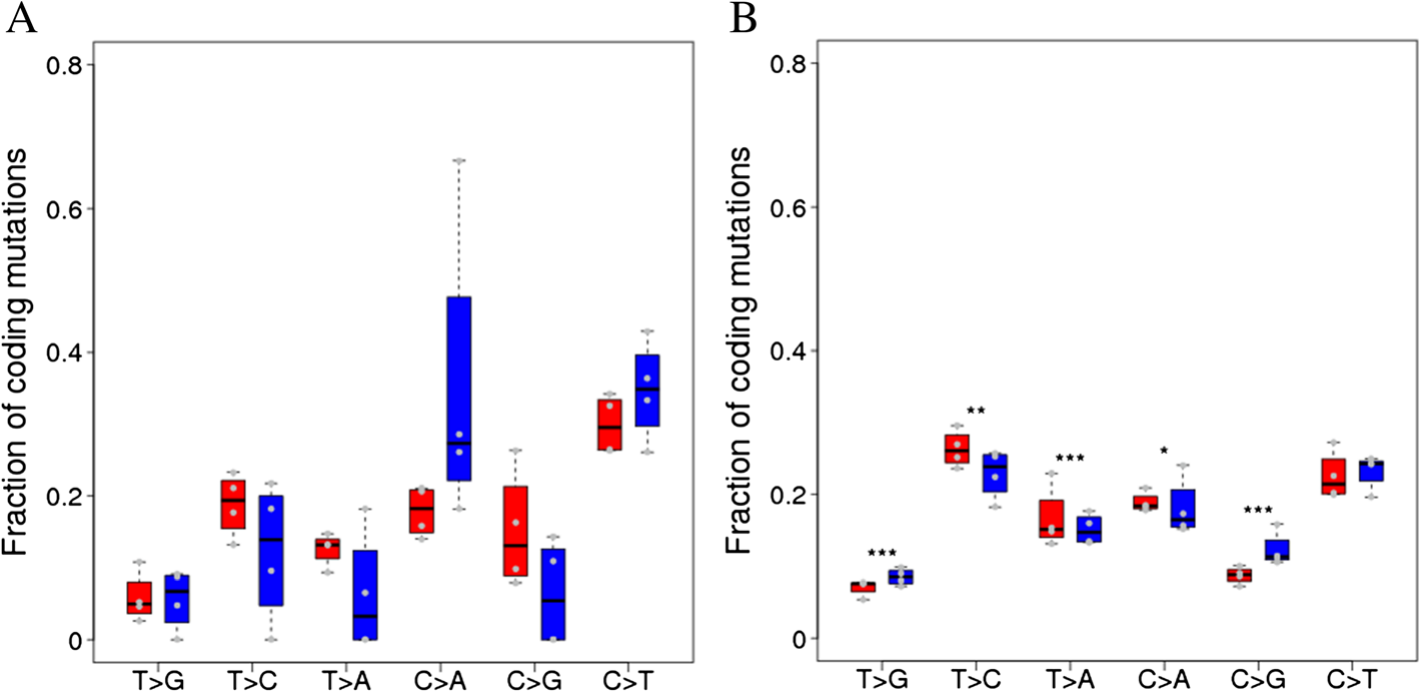
**Comparison of mutational classes between primary tumors and metastasis samples. (A)** The transition and transversion categories of coding mutations are classified from primary tumor and metastasis-specific groups. **(B)** Fraction of overall mutations spanning the transition and transversion categories are concluded in primary tumor and metastasis-specific groups. Red boxes represent primary tumor and blue boxes represent metastasis. Asterisks represent significant difference between primary tumor and metastasis (*, 1e-3<*P* ≤ 5e-2; **, 1e-10<*P* ≤ 1e-3; ***, *P* ≤ 1e-10).

An overview of the mutation spectrum shows that the C:G > T:A transition rates within the coding regions were the highest, i.e. 28.96% in primary tumors and 32.10% in metastases (Figure 3A and Additional file 1: Table S2D), both of which were higher than those observed at the whole-genome level (21.71% vs. 22.22%; Figure 3B). In addition, the second highest mutation type was C:G > A:T transversion (19.40% vs. 18.27%) which was consistent with the notion that C:G > A:T transversion is associated with HBV-positive HCC [12]. Throughout the genetic regions, less mutations of C nucleotides to other nucleotides were located in the CpG islands, whereas more C:G > T:A mutations were found in the non-CpG island regions (Additional file 1: Table S2B and S2C), which may be explained by the fact that cytimidines outside of the CpG islands are easily methylated and mutated. In total, the transversion rate was significantly higher in metastases than in primary tumors (52.65% vs. 54.70%; *P* = 0.0003531) (Additional file 1: Table S2E). Among 6 classes of transition and transversion mutations, we observed an average of 2.39% and 4.87% increases in T:A > G:C (*P* = 8.46e-16) and C:G > G:C (*P* < 2.2e-16) transversion, respectively, in metastasis-specific mutations (Figure 3B and Additional file 1: Table S2E), which may be caused by microenvironment seeding or effects of chemotherapy [13].

### Mutant allele frequency

**Figure 4:**
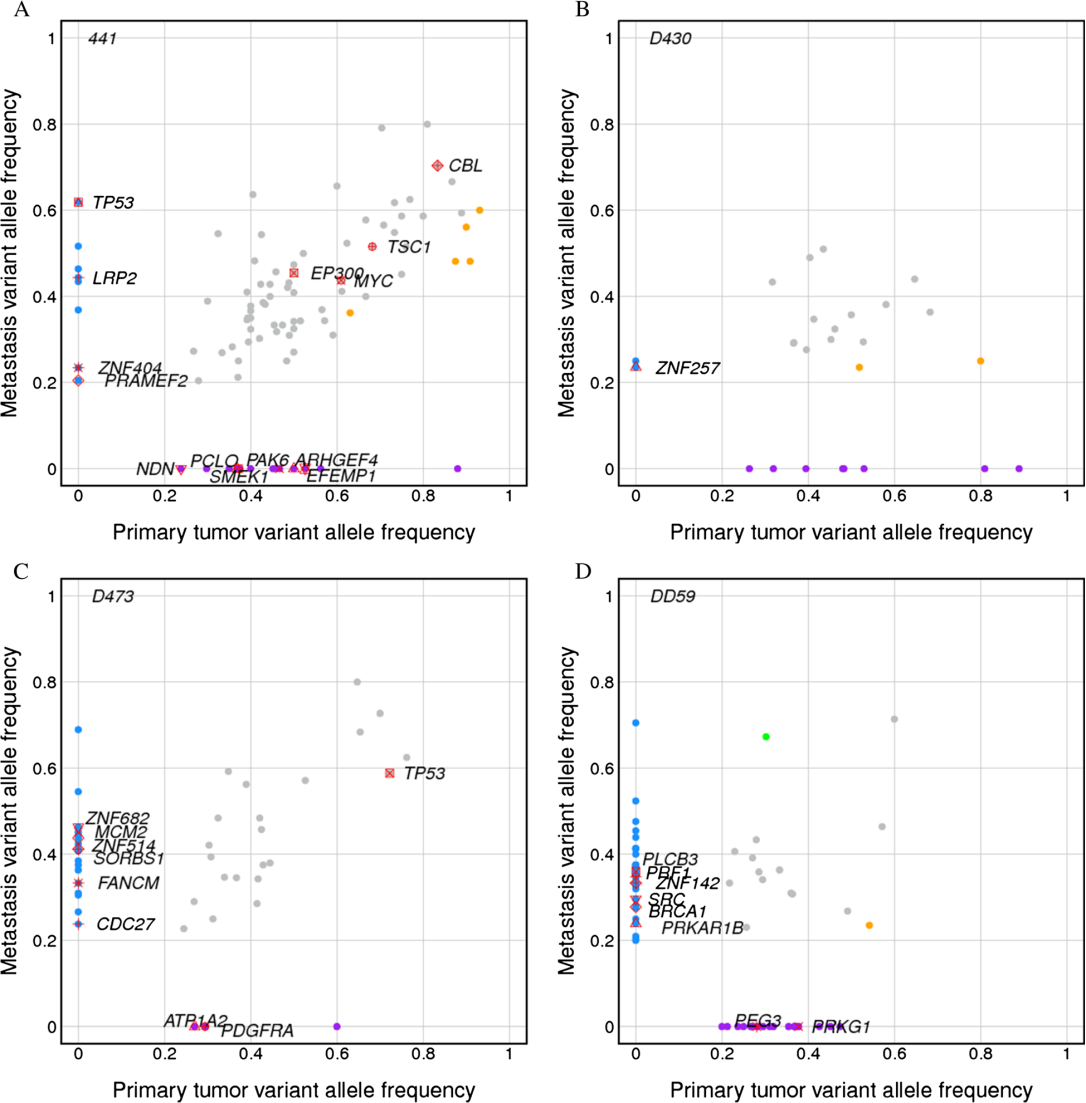
**Comparison of nonsynonymous and splice mutation frequency between primary tumors and metastases.** Each point represents a somatic single-nucleotide variant (SNV). Allele frequency enrichment was tested using the Fishers’ exact test. Gray dot, shared mutations; orange, significantly enriched mutations in primary tumors; green, significantly enriched mutations in metastases; purple, primary tumor-specific mutations; blue, metastasis-specific mutations. SNVs in cancer genes or genes located in cancer-related pathways are labeled with the corresponding gene names. SNVs in each of the 4 cases show in plots **A** to **D** separately.

To delineate possible genetic evolution during metastasis, we carried out an in-depth examination of the mutations of paired primary and metastasis samples. While a large portion of SNVs were shared by both stages, significant differences were observed between the allele frequency of nonsynonymous and splicing-site SNVs in cancer related genes (i.e. the genes in cancer census gene database [14] (Figure 4; Fisher’s exact test). For instance, a nonsense mutation (E304*) in TP53, which regulates TAT binding, was found in both primary tumors and metastases of case D473 (Figure 4C and Additional file 1: Table S3), whereas a deleterious missense mutation in the DNA-binding domain of TP53 (N131D) was only detected in metastases of case 441 (Figure 4A and Additional file 1: Table S3). In addition, we found the similar SNV frequency in a set of oncogenes and tumor suppressors, such as CBL and MYC, between primary tumors and metastases. Non-silent mutations of BRCA1, PRF1, and other cancer genes were detected solely in the metastasis stage (Figure 4 and Additional file 1: Table S4). Moreover, we found 9 mutations with significantly different allele frequency between in primary tumor and in metastasis. The 8 mutations are located in genes KIAA1377, SESN3, MYH4, ZNF613, C5, SPEN, MYO9A and DNM3. Of which, MHY4 is involved in tight junction pathway, SESN3 plays a role in p53 signaling pathway, SPEN is involved in Notch signaling pathway which regulates transcription. Intriguingly, multiple mutations of zinc finger proteins (ZNF) were detected in the metastasis samples; among these, metastasis-specific genetic mutations of ZNF257 and ZNF682 have been reported to be upregulated in metastatic cell lines (lung and bone), relative to the parental line of breast adenocarcinoma [15]. Overall, mutations in genes that are involved in the cell cycle, apoptosis, DNA repair, and transcription regulations were enriched in the metastasis stage (Additional file 1: Table S10B). On the basis of the allele frequency distributions, we can focus on the metastasis-specific genetic alterations to further investigate its underlying mechanisms of lung metastases in HCC. Dissection of the somatic mutations in primary tumors and metastases shows that the affected genes are involved in the focal adhesion and gap junction pathways (Additional file 1: Tables S3, S10A and S10B). In the future, high-depth sequencing based on our candidate list may help explore the subclones or key drivers in metastasis. The 66 somatic mutations were genotyped by Sanger sequencing, and 60 mutations were validated (6 failed to be validated) (Additional file 1: Table S11).

In addition to the mutation frequency, we examined whether metastasis-derived mutations occur at certain genomic regions. However, we did not find any obvious hypermutation region in any of the 4 trio samples (Additional file 2: Figure S4). To further investigate the inter-somatic mutation distance, we compared the inter-somatic mutation distances to the simulated null distributions. The results show that the observed somatic mutation distances (<100 kb) were spaced more closely rather than randomly spotted (Additional file 2: Figures S2 and S3) and that the mutation events occurred in a similar manner in both primary and metastatic tumors (Additional file 2: Figure S5). We also analyzed the inter-somatic mutation distances across different cancer types, of which data was derived from public databases (Materials and methods), such as liver cancer, lung adenocarcinoma, and prostate cancer. Interestingly, we found that a specific tumor type may develop its own intrinsic mutation distance (Additional file 2: Figure S5). Together, our findings suggest that the scattered small regions with dense mutations are present in the cancer genomes and that specific cancer types are associated with their own mutation distance.

### Short insertions and deletions

We predicted 2,514-4,925 and 4,366-7,968 indels in primary tumors and metastases, respectively, including a total of 25/34 (primary tumor/metastasis) events affecting protein coding (18/24 frameshift and 7/10 in-frame) in 42 genes (Additional file 1: Table S2F). Specifically, a frameshift insertion in the tumor suppressor RB1 was detected in both primary tumors and the matched metastases, whereas a metastasis-specific frameshift deletion was detected in the oncogene TFEB. In addition, a frameshift insertion in WNT5A and an in-frame deletion in DAAM2 were only detected in metastases; both WNT5A and DAAM2 are involved in the Wnt signaling pathway (Additional file 1: Table S10C).

### Copy number alteration

Copy number alterations (CNAs) in primary tumors and metastases of 4 cases were detected by comparing with their respective matched normal samples. From primary tumors to metastases, the lengths of CNAs were changed, but the total sizes of CNAs were not significantly different (Additional file 1: Table S2G). As indicated by the CNA plots, primary tumors and their matched lung metastases generated similar global CNA patterns across 4 pairs (Additional file 2: Figure S6). The copy numbers across the genomes between primary tumors and metastases were found to be positively correlated with each other with correlation coefficient >0.5 for all 4 cases (Additional file 2: Figure S7), consistent with the conclusion that the genetic characteristics of metastases mostly originate from primary tumors. The copy numbers of CNA events were altered from primary tumors to metastases, and these CNAs were annotated to corresponding genes (Additional file 1: Table S7). Specifically, many CNAs with common regions but different copy number between primary tumor and metastasis are abserved in cases DD59 and D473. Such a pattern corresponds to the low percentage of somatic SNVs preserved in metastases (Figure 2). We found 26 CNA regions (26 deletions and no amplification) in primary tumors and 235 CNA regions (173 deletions and 62 amplifications) in metastases. Notably, 11 of the 26 deletions observed in primary tumors overlapped with 11 deletions in metastases (Additional file 2: Figure S7 and Additional file 1: Table S2H). The deletions detected in both primary tumors and metastases were mainly located in 1p, 4q, 8p, 9p, 9q, 16p, and 21q (Additional file 1: Table S7). By categorizing tumor-specific CNA regions in metastases, we found that alterations of oncogenes and tumor suppressors, for instance, tumor suppressor PMS2 and oncogenes TFPT, ZNF331, and others, may be linked to metastatic progression (Additional file 2: Figure S7 and Additional file 1: Table S7). In addition, frequent somatic copy number loss in the region spanning the tumor suppressor genes, CDKN2A and CDKN2B, was also observed across metastasis samples (Additional file 1: Table S7) [8].

### Structural variation

We used CREST to detect structural variants (SV) in sequencing data [16]. We identified 65–221 and 60–232 SVs in primary tumors and metastases, respectively. Indeed, the large portion of SVs in primary and metastatic tumors overlapped with each other (Figure 1). We identified 30 translocations that were only in metastases, 8 translocations that were only in primary tumors, as well as 30 overlapped translocations (Additional file 1: Table S2I). Some of the oncogenes and tumor suppressors, such as TPM3 and ZNF331, which were located in the breakpoints of somatic SVs, were detected in our study (Additional file 1: Table S6). Specific mutated genes that were identified in tumorigenesis pathways are summarized in Additional file 1: Table S10. Specifically, distinct mutated genes in metastasis, CNTNAP2 and PDCD1LG2, are the components of the cell adhesion pathway. The overexpression of CDH13 and AKAP13A, both of which were found to be truncated in our study, have been reported to correlate with a higher risk of late recurrence of hepatocellular carcinoma [17,18]. In addition, the CDH13 and PDCD1LG2 genes located at the breakpoints of an inter-chromosome translocation may cause the CDH13-PDCD1LG2 fusion.

### Potential regulation pathways of tumor progression

To identify genes as “driver” somatic mutations in either primary tumorigenesis or metastasis process, the genes with significant higher nonsynonymous mutation rates against a background were estimated [19]. Tumor suppressors PRF1 and BRCA1, as well as oncogene TFEB, all of which may play an important role in cancer cell survival during the progress of metastasis, were predicted as driver genes only in metastases. WNT5A, PLCB3, and DAAM2, which can activate or inhibit canonical Wnt signaling, were predicted as metastasis-specific driver genes (Additional file 1: Table S10F). TP53, a tumor suppressor that was previously reported to be associated with liver cancer [14], was also weighted as a driver gene and is recurrently mutant in metastases (Additional file 2: Figure S2). In addition, we identified that shared mutations between primary tumors and metastases were significantly enriched in the pathways of bladder cancer, pancreatic cancer, non-small cell lung cancer, thyroid cancer, and melanoma, which implies that the general progress of different tumor types may be driven by several same mutations. The mTOR signaling pathway and some others were identified to be significant only in primary tumors (Additional file 1: Table S9A).

**Figure 5:**
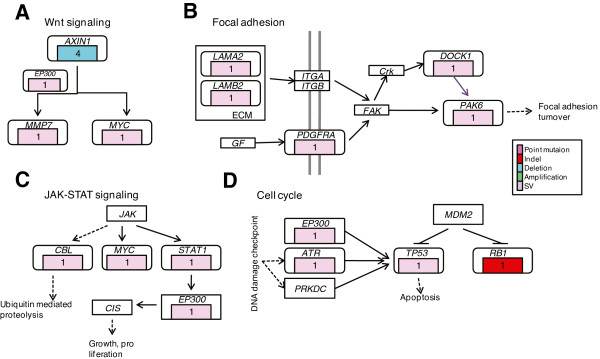
**Significantly altered pathway in primary tumor.** Pathway analysis for primary tumor mutant genes identified significant genomic alteration in several cancer pathways, including **(A)** Wnt, **(B)** focal adhesion, **(C)** JAK-STAT and **(D)** cell cycle pathways. Alterations included somatic SNVs, Indels, CNAs and SVs. Alteration recurrence was referred as number of cases harboring a mutant gene. Alteration types were represented by different colours.

**Figure 6:**
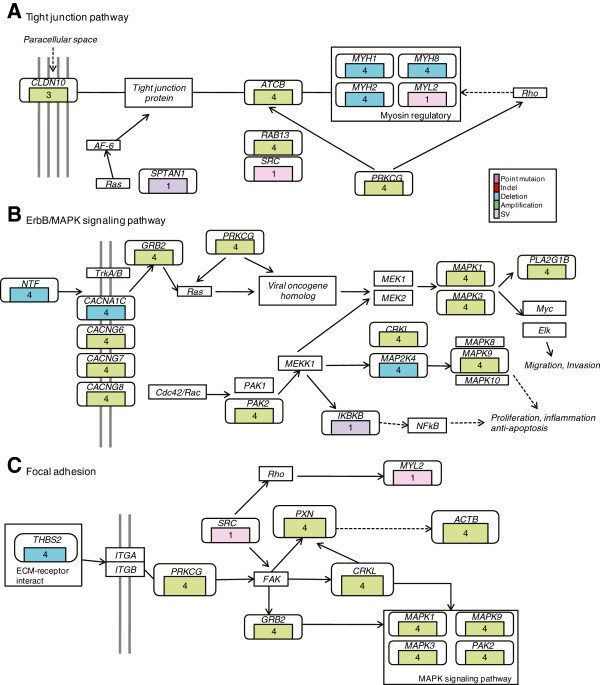
**Significantly altered pathway in metastasis.** Pathway analysis for metastasis-specific mutant genes identified significant genomic alteration in multiple cancer/cell migration relevant pathways, including **(A)** tight junction, **(B)** ErbB/MAPK and **(C)** focal adhesion pathway. Alterations included somatic SNVs, Indels, CNAs and SVs. Alteration recurrence was referred as number of cases harboring a mutant gene. Alteration types were represented by different colours.

By integrating altered genes in primary tumor and specifically altered genes in metastasis from four cases, we concluded several cancer and cell migration relevant pathways. Altered pathways in primary tumor were Wnt, JAK-STAT, cell cycle and focal adhesion pathways (Figure 5), which function in tumor initiation and development. On the other hand, metastasis-altered pathways included tight junction, focal adhesion and ErbB/MAPK pathways (Figure 6). Tumor cells in metastasis carried alterations enabling migration and circulation and derived additional alterations, which may endowi their ability to clonalize in distant organ and grow as primary tumor cells [20].

## Conclusion

In summary, genetic alteration patterns are very similar between primary tumor and metastasis of 4 cases (Figure 1, Additional file 2: Figure S2 and Table 2). For somatic SNVs, we concluded that 38.88-78.49% mutations detected in primary tumors were preserved in matched metastases (Figure 2). Also copy number along tumor genomes are very close correlated between primary tumors and matched metastases, with correlation coefficients range of 0.51-0.82 (Pearson correlation coefficient) for 4 cases (Additional file 2: Figure S7). In addition to SNVs and CNAs, SVs have the similar pattern across 4 pairs with 30.21-74.07% SVs detected in primary tumors preserved in metastases. From all above, we can approximately conclude that genetic feature in primary tumor and metastasis of case 441 are the most similar, and case D430 ranks the second most similar and then case D473 and case DD59. Specifically on clinical features, case 441 had been performed simple treatment with only liver lobectomy and without any recurrence during primary tumor developing to metastasis. Case D430 also underwent simple segmentectomy, however, developed recurrence after metastasis detected. Both cases of D473 and DD59 underwent TACE before metastases were detected, which may cause point mutations [13], consistent with that percentages of common point mutations between primary tumor and metastasis are low for these two cases (Figure 2).

**Table 2:** Similarity of genetic alterations in primary tumors and matched metastases

**Statistics**	**441**	**D430**	**D473**	**DD59**
% of met_SSMs preserved in primary tumor	78.49%	72.55%	53.26%	38.88%
% of met_Indels preserved in primary tumor	63.64%	100.00%	57.14%	28.57%
Correlation coefficients of copy numbers between primary tumor and metastasis	0.69	0.51	0.82	0.53
% of met_SVs preserved in primary tumor	74.07%	43.08%	67.87%	30.21%

## Discussion

HCC is the fifth most common cancer and the third most common cause of cancer-related deaths worldwide. It has a high prevalence in Asia, including Korea, due to endemic hepatitis B virus infection. numerous clinical trials using cytotoxic chemotherapy or targeted agents have failed, except sorafenib [21]. While intrahepatic recurrence is most common, lung metastases are the most common extrahepatic spread and account for up to 40% of metastatic HCC [22]. survival of patients with metastatic HCC is usually less than 1 year [23]. Recently, prolonged median survival durations have been reported from 6.4 months to 40.7 months following metastasectomies [24]. However, despite advances in therapeutic strategies, extrahepatic metastasis is still a major impediment to better prognosis in HCC patients.

In this study, we employed whole-genome sequencing to profile somatic mutations and structural variations in primary HCC (HBV+) and their matched metachronous lung metastases. Several recent studies on HCCs of different etiologies (HBV, HCV, and alcohol) using whole-genome and exome sequencing have not only confirmed previously known mutations of β-catenin and TP53 but also identified novel genetic alterations in genes that are involved in epigenetic regulation, such as ARID1A, ARID1B, ARID2, MLL, and MLL3 [12]. Wnt/β-catenin, p53 signaling, cell cycle, and chromatin remodeling have recently emerged as dominant cancer pathways in primary HCC [12]. To the best of our knowledge, this study is the first to examine the genomic profiles of matched pairs of primary and metastatic HCC by whole-genome sequencing.

In summary, comparison of structural variations between primary and metastatic tumors shows very similar and largely overlapped mutated segments (Figure 1). In addition, CNVs in primary and metastatic pairs were found to be closely related, and 38.88-78.49% of SNVs detected in primary HCC tumors were also detected in lung metastases. These preservations in genomic profiles from liver primary tumors to metachronous lung metastases indicate that the genomic features during tumorigenesis may be retained during metastasis.

Despite similarities between primary and metastatic pairs, a few somatic mutations were detected only in metastatic tumors. Multiple mutations of zinc finger (ZNF) genes were detected in the metastasis samples, and mutated genes that are involved in apoptosis and transcription regulations were enriched in metastases (Additional file 1: Table S10B). The ZNF family represents a large group of proteins involved in various aspects of transcriptional regulation [25]. It has been reported that there were almost twice as many ZNF mutated genes in the HBV-positive hepatitis [26]. In our study, a total of 5 ZNF, namely ZNF257, ZNF682, ZNF404, ZNF514, and ZNF142, were mutated in lung metastases from HCC (Additional file 1: Table S10B). Among these, ZNF257 and ZNF682 were previously reported to be upregulated in lung and bone metastases, relative to the primary breast adenocarcinoma, and were speculated to be putative metastasis genes [15]. In addition, ZNF mutations were found in head and neck squamous cell carcinoma (HNSCC); therefore, the genetic changes to these transcriptional regulators have been implicated in the development of HNSCC [27]. Nevertheless, the functional role of ZNFs in HCC tumorigenesis is yet to be defined.

In addition, mutations in known cancer gene suppressor TP53 were also detected in our study samples, including a nonsense mutation (E343*) in the regulator of TAT binding and a missense mutation (N131D) in the DNA-binding domain, both were previously reported by COSMIC (http://cancer.sanger.ac.uk/cancergenome/projects/cosmic/) (Additional file 1: Table S3).

Notably, a frameshift insertion in WNT5A and an in-frame deletion in DAAM2 were detected only in lung metastases; both WNT5A and DAAM2 are involved in the Wnt signaling pathway. Recent genome sequencing studies have revealed that the WNT signaling pathway is the most frequently altered oncogenic pathway in primary HCC [12]. In addition to the high prevalence of CTNNB1 mutations, genetic alterations of other components, such AXIN1, AXIN2, and APC genes that encode proteins containing the destruction box, also occur at a lower frequency. Wnt5a, which is up-regulated in poorly differentiated and highly motile mesenchymal-like HCC cells, has been suggested to play a role in tumor progression by inducing epithelial mesenchymal transition [28]. In line with this, Wnt5a up-regulation was significantly shown to enhance migration, proliferation, and invasiveness in pancreatic cancer cells *in vitro*[29]. Importantly, Wnt signaling has been implicated in the activation of HCC-initiating cells [30,31]. While Wnt5a has been described as a tumor promoter in melanoma, gastric, pancreatic, and prostate cancers, it was suggested to be a tumor suppressor in HCC, neuroblastoma, leukemia, colon, and thyroid cancers [32]. The functional significance of Wnt5 aberrations in tumorigenesis of lung metastases from HCC primary tumors should be evaluated in subsequent studies.

Synthetically, primary tumor harbored more alterations located in Wnt, cell cycle, JAK-STAT pathways (Figure 5), which plays an important role in encoding protein molecular emphasizing the cell proliferation, apoptosis and inflammation, as well as focal adhesion pathway. Focal adhesion pathway has been implicated in the cell migration and initiated metastasis [33]. For metastasis, specific alterations arose in tight junction and focal adhesion pathways (Figure 6), which enhanced the cell migration mechanism.

In this study, for the first time, the whole-genome sequencing profiles of primary HCC and paired lung metastases were compared. We found similar genomic alterations between them, with a few mutations that were found specifically in lung metastases, which may explain the clinical observation that both primary and metastatic tumors are usually sensitive or resistant to the same systemic treatments.

## Methods

### Tumor specimens and ethical statement

A total of 4 pairs of fresh frozen tumors and adjacent non-tumor liver tissues containing no necrosis or hemorrhage were collected from primary HCC patients treated with curative hepatectomy at the Samsung Medical Center, Seoul, Korea. Lung tumor specimens were collected at the time of lung metastasectomy. This study was approved by the Institutional Review Board of Samsung Medical Center, Seoul, Korea.

### DNA

Genomic DNA was extracted from sliced tissue specimens using the QIAamp DNA Mini Kit (Qiagen, Hilden, Germany), respectively.

### Library construction and whole-genome sequencing

Qualified genomic DNA samples were randomly fragmented by Covaris. After ligation of sequencing adapters to both ends of resulting fragments, successfully adapter-ligated DNA fragments were purified, and those with an insert size of approximately 500 bp were selected by electrophoresis. Insert fragments with correct sizes were harvested and prepared for cluster amplification by cBot (Hiseq 2000).

The qualified DNA library was then loaded on Hiseq2000 for high-throughput sequencing, to meet required sequencing depth with 90-bp paired-end reads. Raw image files were read by the Illumina’s base calling software with default parameters, and raw data were collected.

### Read mapping and somatic variant detection

Sequencing reads from primary and metastatic tumors, as well as matched normal tissues, were gap-aligned to the hg19 reference genome by BWA program, followed by removal of PCR duplicates. Somatic variants in primary and metastatic tumors were detected by comparing tumor reads to those of matched normal samples. Somatic mutations in tumors were predicted by SOAPsnv (http://soap.genomics.org.cn/index.html) using a sensitive score threshold. Next, *p* values were calculated using Fisher’s exact test for all putative mutation sites, based on the distribution of read support with high stringent specificity and sensitivity [11]. For mutations present in dbSNP (which are usually germline mutations), we removed potential germline mutations existing in database of dbSNP132 for the downstream analysis. Subsequently, we annotated potential somatic SNVs by mapping them to RefGene (http://refgene.com/) using an in-house annotation program.

Indels in tumors were examined through gapped alignment not allowing gap located 5 bp to either end of the mapped reads [11]. From the indel pool, somatic indels were detected by filtering SNV which was also detected in adjacent normal samples. Somatic indels with ≤5 supporting reads were filtered for high confidence.

We used in-house software based on the read-depth method to infer copy number variation in primary and metastatic tumors. Copy number variants were called when the copy number in tumors was ≤0.75- or ≥1.25-fold of that in corresponding normal samples. To infer recurrently amplified or deleted genomic regions, we detected the focal copy number segments (≤1Mbp) by re-implementing the GISTIC algorithm [34] using copy numbers in 1-kb windows, instead of SNP array probes as markers. G-scores were calculated for genomic and gene-coding regions, based on the frequency and amplitude of amplification and deletion affecting each gene, respectively. A significant CNV region was defined as having an amplification G-score >0.08 or a deletion G-score <0.09, corresponding to a *p*-value threshold of 0.05 from the permutation-derived null distribution [34].

We further applied CREST [16] to whole-genome DNA sequence data obtained from primary and metastatic tumors to identify candidate structural variants (SV) at base-pair resolution.

### Prediction of driver genes

Driver genes were predicted using in-house software by re-implementing the algorithm in which driver genes are the genes with a significant higher non-silent mutation (nonsynonymous/splice/coding indel) rate against a background mutation rate estimated from collected silent mutations [19]. Similarly, driver pathways were predicted by computing the significance of enrichment of non-silent mutations in a certain pathway against background.

### Pathway enrichment analysis

Pathway enrichment analyses of genes harboring somatic SNV, small coding indel, CNV, or SV were performed using the Gene Set Enrichment Analysis [35,36].

### Genomic distribution of somatic mutations

We computed the inter-somatic mutation distance within individuals by defining the inter-somatic mutation distance as the distance from a somatic mutation to the very next one. We computed null distributions by randomly sampling positions from the genome (excluding assembly gaps), while maintaining the number of somatic mutations per chromosome [37]. Using the Kolmogorov–Smirnov test, we calculated the differences between real distributions and corresponding null distributions and found that inter-somatic mutation distances were significantly smaller than expected (*p* < 0.05). The distribution of inter-somatic mutation distance in primary and metastatic tumors of sample D473 were compared to the distributions of inter-somatic mutation distances in HCC and other types of tumors, using published data downloaded from ICGC (ftp://data.dcc.icgc.org/version_11/). We used 2 lung adenocarcinoma samples, TCGA-05-4382-01A-01D-1265-08 and TCGA-05-4424-01A-22D-1855-08, from TCGA (http://cancergenome.nih.gov/), 2 prostate cancers, CPCG_0099_Pr_P_P1 and CPCG_0184_Pr_P_P4, from OICR (http://oicr.on.ca/), 2 liver cancers, HCC122T and RK020_C01, from NCC (http://www.ncc.go.jp/en/), and 1 liver cancer sample (sample 180) sample, sample180, from a collaboration HCC project of which some results have been published [11].

### Data access

Sequencing data for this publication has been deposited in NCBI Short Reads Archive and is accessible through accession number SRA076160.

Written informed consent was obtained from the patient (who was not deceased at the time of writing or waived by the IRB if deceased) for the publication of this report and any accompanying images.

## Abbreviations

HCC: Hepatocellular carcinoma; SNVs: Single-nucleotide variants; SVs: Structural variations; CNAs: Copy number alterations; SSMs: Somatic SNV mutations; NGS: Next-generation sequencing; TACE: Transcatheter arterial chemoembolization; COSMIC: Catalogue of somatic mutations in cancer; ICGC: International cancer genome consortium; TCGA: The cancer genome atlas; OICR: Ontario institute for cancer research; NCC: National cancer center.

## Competing interest

The authors declare that they have no competing interests.

## Authors’ contributions

JL, HL, JGK, CKP initially designed this study. LO, YZ performed research and analyzed data. LO, JL, CKP, JGK, HL, MM, YS, ZG and HZ designed the research and reviewed the manuscript. LO, JL, JGK, HL, CKP, MM, YS, ZG wrote the paper. All authors read and approved the final version of the manuscript for publication.

## Pre-publication history

The pre-publication history for this paper can be accessed here:

http://www.biomedcentral.com/1755-8794/7/2/prepub

## Supplementary Material

Additional file 1 Contains tables of summaries of variants, variant lists, gene lists and pathway lists. Click here for file

Additional file 2 Contains figures of clinical images and variants. Click here for file
