# Supplementary material for: Whole-genome sequencing of matched primary and metastatic hepatocellular carcinomas
Source: BMC Med Genomics. 2014 Jan 9;7:2. doi: 10.1186/1755-8794-7-2 (PMC3896667; doi:10.1186/1755-8794-7-2)

**Supplementary Information**

**Legends of supplementary figures**

**Figure S1. Pathology pictures of 4 individuals.**

**Figure S2. Common variants between primary tumor and metastasis.** The overlap of somatic SNVs, Indels and SVs between primary tumor and metastasis for the four patients.

**Figure S3. Recurrently mutant genes among patients.** Recurrent genes were defined as genes which have somatic SNV or Indel in at least 2 patients. Number in the lattice represents number of mutations for the specific genes in the corresponding patient tumor.

**Figure S4. Rainfall plots for primary tumors and the matched metastases of the 4 samples.** Mutations were ordered sequentially on chromosomes and their distances to the very next mutation were plotted, coloring by class of substitution. The MAF panel in the bottom shows the tumor allele frequencies (from 0 to 1) for those somatic SNVs.

**Figure S5. Nonrandom distribution of somatic mutations in genomes.** Plots of the observed distribution of inter-somatic mutation distances within individuals and the expected distribution based on a random mutation model. Differences are statistically significant by the KS test with p values < 0.05. The distribution of inter-somatic mutation distance from primary tumor (circle points) and metastasis (triangle points) of sample D473 are compared to distributions of inter-somatic mutation distances of HCC and other types of tumors using data from ICGC.

**Figure S6. CNAs plots along genomes for primary tumors and the matched metastases of the 4 samples.** The upper panel shows the copy number ratio(compared to primary tumor adjacent tissue) of primary tumor and the lower panel shows the copy number ratio(compared to primary tumor adjacent tissue) of metastasis.

**Figure S7. CNA ratio between primary tumors and metastases of the 4 samples.** The X axis indicates the primary tumor’s log2 copy number ratio relative to the adjacent tissue. Y axis indicates the metastasis’s log2 copy number ratio relative to the primary tumor adjacent tissue. [Spearman's rank correlation coefficient](http://203.208.46.148/url?sa=t&rct=j&q=spearmans&source=web&cd=1&ved=0CC8QFjAA&url=%68%74%74%70%3a%2f%2f%65%6e%2e%77%69%6b%69%70%65%64%69%61%2e%6f%72%67%2f%77%69%6b%69%2f%53%70%65%61%72%6d%61%6e%27%73%5f%72%61%6e%6b%5f%63%6f%72%72%65%6c%61%74%69%6f%6e%5f%63%6f%65%66%66%69%63%69%65%6e%74&ei=pB-HUYypGaqjiAfanID4Cg&usg=AFQjCNGG7VyKUBSAQMZLOG9qlkAld40y3Q&cad=rjt) and Pearson correlation coefficient were calculated. This figure shows the copy number gain or loss of metastasis against that of primary tumor. The ratio thresholds for copy number gain and copy number loss of metastasis against copy number of primary tumor are 1.25 and 0.75 respectively. The red points are the CNAs overlapped with CNA regions, and the blue points represent CNAs located in cancer genes.

**Supplementary tables S1 to S12** – Please refer to individual worksheet in excel file

Table S1. Clinico-pathological data of 4 patients.

Table S2. Summary for WGS and predicted somatic variants.

Table S3. List of all protein-altering somatic SNVs identified in the cohort.

Table S4. List of genes with protein-altering somatic mutations.

Table S5. List of protein-altering somatic small indels identified in the cohort.

Table S6. List of all somatic SVs identified in the cohort.

Table S7. List of significant CNV regions in the cohort.

Table S8. Driver genes prediction results.

Table S9. Driver pathways prediction results.

Table S10. Pathways summary.

**Supplementary figures**

**Figure S1**

**
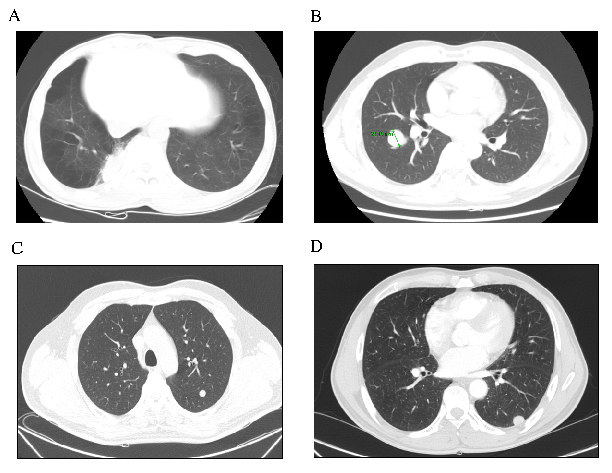
**

**Figure S2**

**
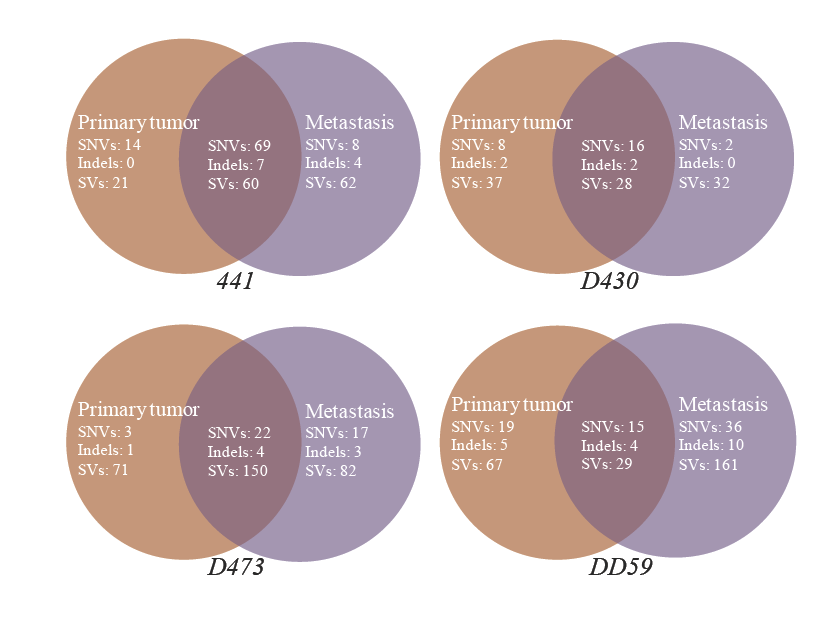
**

**Figure S3**

**
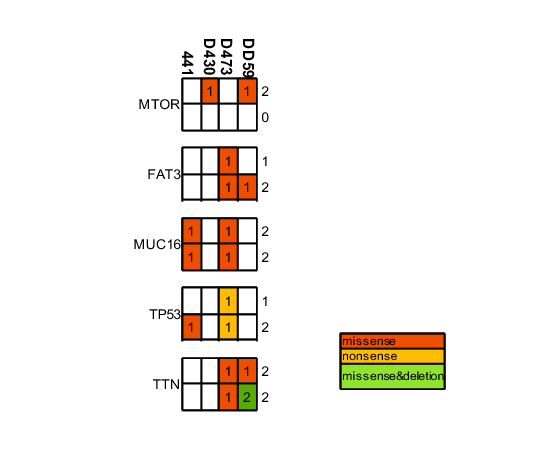
**

**Figure S4**

**
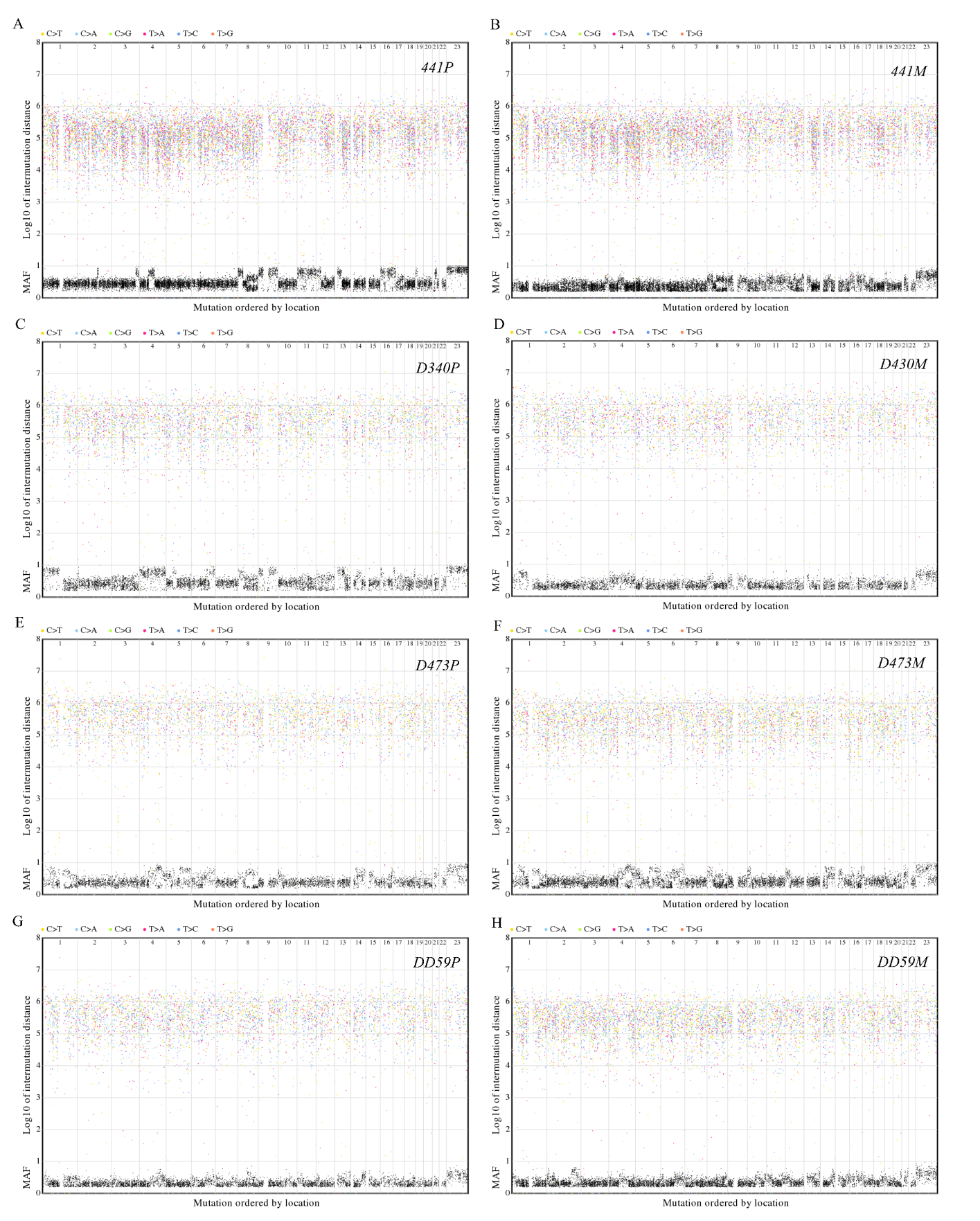
**

**Figure S5**

**
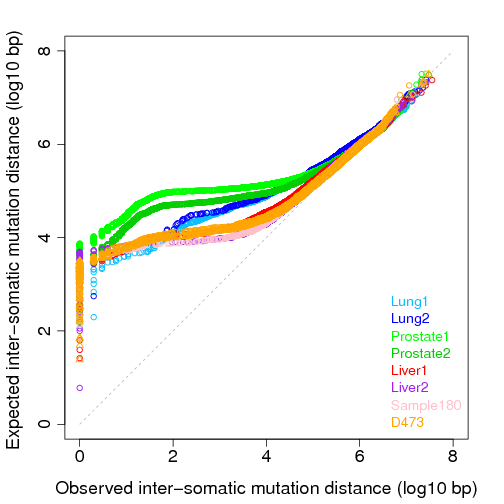
**

**Figure S6**

**
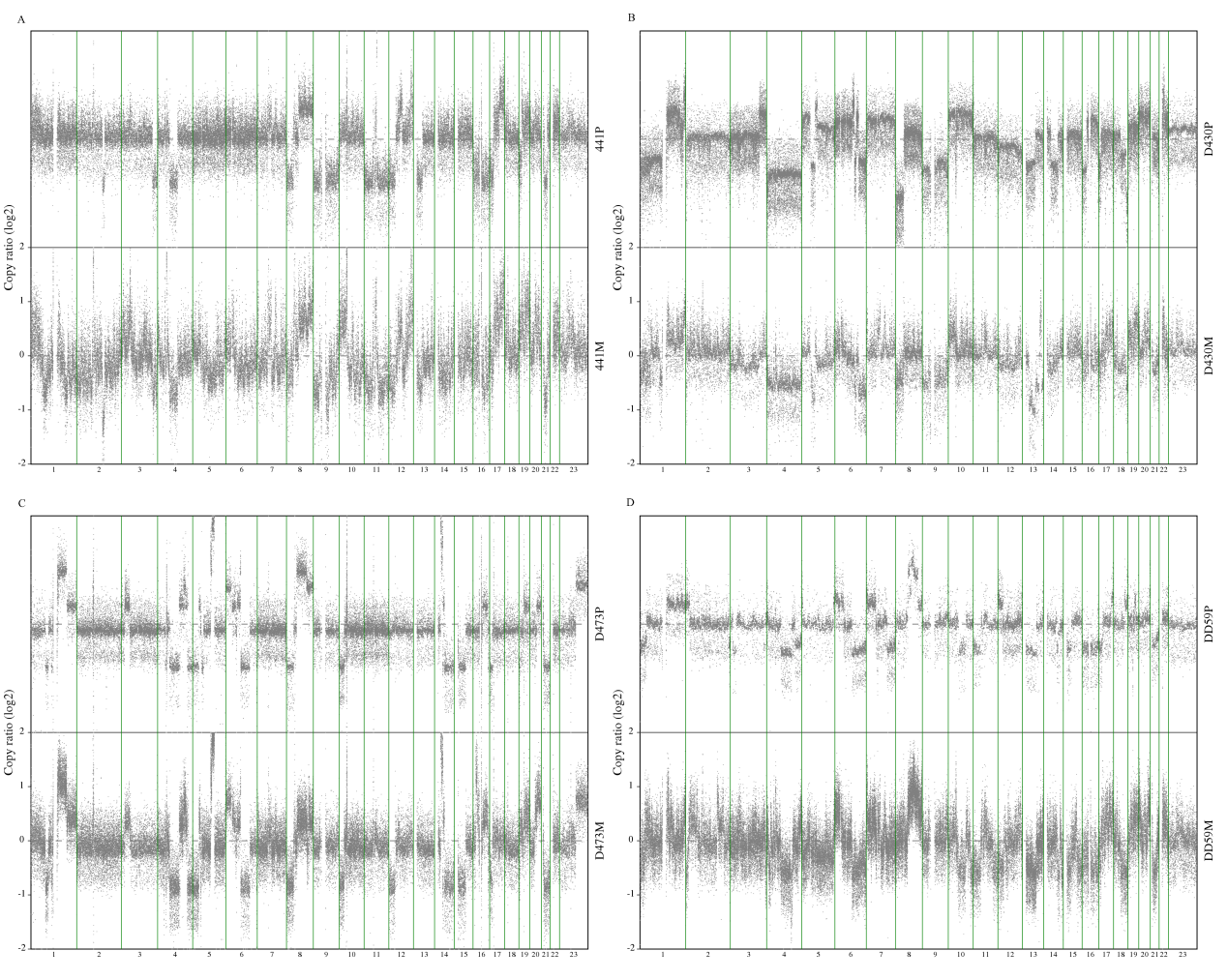
**

**Figure S7**


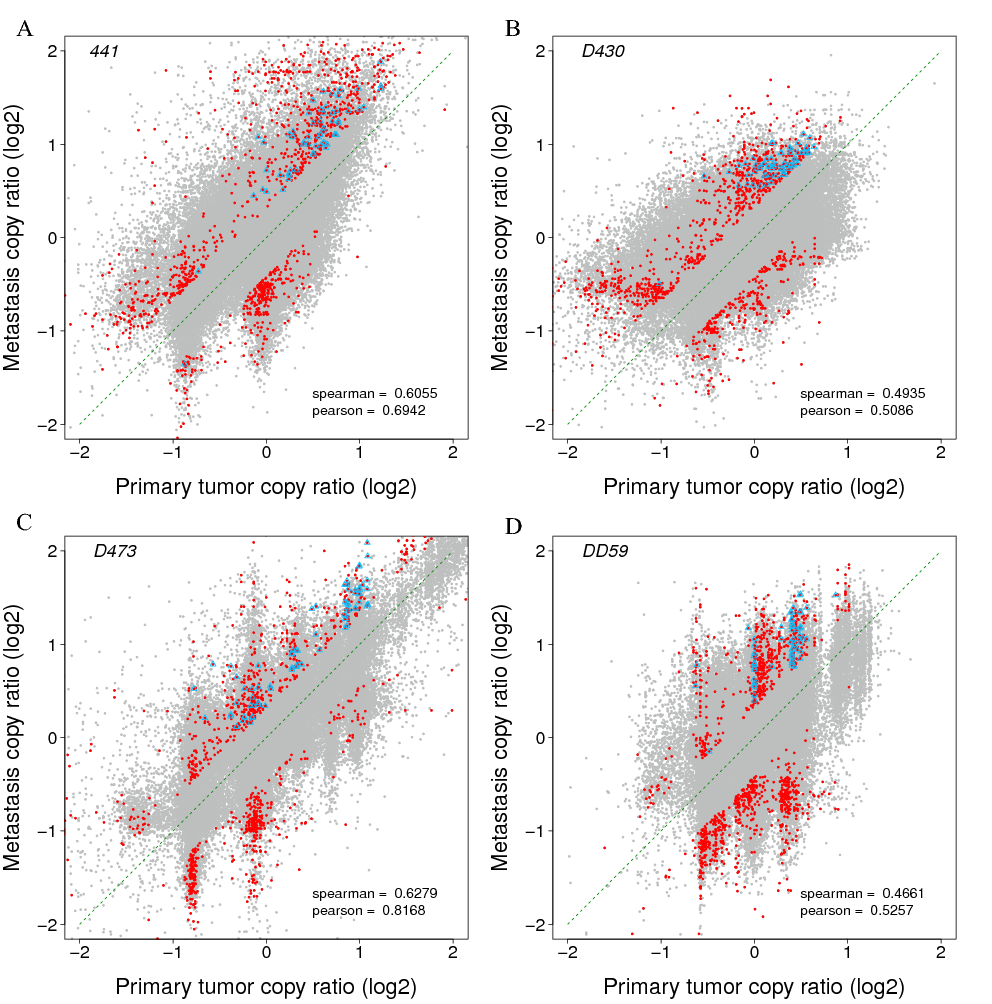

Supplement: Additional file 2 — Contains figures of clinical images and variants. [file 1755-8794-7-2-S2.docx]
